# HLA-DQB1-AS1 Promotes Cell Proliferation, Inhibits Apoptosis, and Binds with ZRANB2 Protein in Hepatocellular Carcinoma

**DOI:** 10.1155/2022/7130634

**Published:** 2022-05-11

**Authors:** Jianwu Long, Longfei Liu, Xiaoyun Zhou, Xianzhou Lu, Lei Qin

**Affiliations:** ^1^Department of General Surgery, The First Affiliated Hospital of Soochow University, Suzhou, Jiangsu 215006, China; ^2^Department of Hepatobiliary Surgery, The Affiliated Nanhua Hospital, Hengyang Medical School, University of South China, Hengyang, Hunan 421002, China

## Abstract

Major histocompatibility complex, class II, DQ beta 1 antisense RNA 1 (HLA-DQB1-AS1) conferred the susceptibility to hepatocellular carcinoma. Sustaining cell growth and resisting apoptosis are two hallmarks of hepatocellular carcinoma. The present study explored the role of HLA-DQB1-AS1 in the proliferation and apoptosis of hepatocellular carcinoma cells and investigated its downstream pathway. Colony formation assay was performed to assess cell proliferation. Cell apoptosis was assessed with the TdT-mediated dUTP nick end labeling method. HLA-DQB1-AS1 deficiency exerts antiproliferative and proapoptotic effects on hepatocellular carcinoma cells. Moreover, based on bioinformatic analysis combined with the results of RNA immunoprecipitation assay, HLA-DQB1-AS1 was revealed to bind with zinc finger RANBP2-type containing 2 (ZRANB2) protein. ZRANB2 was upregulated in hepatocellular carcinoma at a clinical and cellular level. HLA-DQB1-AS1 caused no significant effects on ZRANB2 mRNA and protein expression. ZRANB2 knockdown suppressed cell proliferation and enhanced cell apoptosis of hepatocellular carcinoma. Moreover, ZRANB2 overexpression rescued the anticancer effect of silenced HLA-DQB1-AS1 in hepatocellular carcinoma cells. In conclusion, HLA-DQB1-AS1 promotes cell proliferation and inhibits apoptosis in hepatocellular carcinoma by the interaction with ZRANB2 protein.

## 1. Introduction

It is reported with an estimated 905,677 new cases and 830,180 death cases of hepatocellular carcinoma in 2020 around the world, which account for 4.7% and 8.3% in all cancers, respectively [1]. Hepatocellular carcinoma is a polygenic chronic disease and involves multiple genes and signaling pathways, which affect its treatment. Alcohol, viral infection-induced chronic liver disease, and nonalcoholic fatty liver disease are risk factors for hepatocellular carcinoma [2, 3]. Transarterial chemoembolization, liver resection, ablation, and transplantation are effective for patients in early or intermediate stages [4], while treatment for hepatocellular carcinoma in advanced stage is limited. Sustaining cell growth and resisting apoptosis are two hallmarks of hepatocellular carcinoma [5]. Resistance to apoptosis causes the failure of traditional cancer treatment [6]. Strategies targeting cancer cell apoptosis show the potential for the treatment of hepatocellular carcinoma patients [7]. Our study focused on cell proliferation and apoptosis in hepatocellular carcinoma cells to offer therapeutic insights.

Noncoding RNAs constituting more than 90% of the human genome were increasingly identified and annotated in abundance [8]. Long noncoding RNAs (lncRNAs) with over 200 nucleotides are unable or limitedly able to code proteins [9]. lncRNAs are recognized as essential mediators of various biological processes rather than byproducts of RNA polymerase II transcription or genomic noise [10]. By epigenetic modification, RNA decay, transcription modulation, and posttranscription modulation, lncRNAs control tumorigenesis [11]. Recently, lncRNAs have emerged as therapeutic targets for hepatocellular carcinoma. These lncRNAs can be used as the potential biomarkers for diagnosis and prognosis in hepatocellular carcinoma patients and control the fate of cancer cell proliferation and apoptosis [12–14].

A previous study showed that a causal single nucleotide polymorphism-regulated major histocompatibility complex, class II, DQ beta 1 antisense RNA 1 (HLA-DQB1-AS1) conferred the susceptibility to hepatocellular carcinoma [15]. HLA-DQB1-AS1 has a positive correlation with the survival of patients with lung adenocarcinoma [16] and melanoma [17]. Based on bioinformatic analysis, HLA-DQB1-AS1 exhibits significant upregulation in clinical hepatocellular carcinoma tissues, while its role in the development of hepatocellular carcinoma remains unclear.

In this study, we aimed to explore the effect and underlying mechanism of HLA-DQB1-AS1 in the proliferation and apoptosis of hepatocellular carcinoma cells. We assumed that HLA-DQB1-AS1 regulates hepatocellular carcinoma progression by binding with zinc finger RANBP2-type containing 2 (ZRANB2). The findings of our study may provide clues for the targeting therapy of hepatocellular carcinoma.

## 2. Materials and Methods

### 2.1. Bioinformatic Analysis

ENCORI database (https://starbase.sysu.edu.cn/panGeneDiffExp.php) was used to predict the expression of HLA-DQB1-AS1 and ZRANB2 in the tissue samples of liver hepatocellular carcinoma patients. RBPDB database (http://rbpdb.ccbr.utoronto.ca/) predicted the binding between HLA-DQB1-AS1 and ZRANB2. The GEPIA database (http://gepia.cancer-pku.cn/) was used to predict the relation between ZRANB2 expression and hepatocellular carcinoma patient survival or tumor stage and the expression correlation between HLA-DQB1-AS1 and ZRANB2 in normal tissues or hepatocellular carcinoma tumor tissues.

### 2.2. Cell Lines and Cell Culture

The human hepatocyte cell line LO2 (#MZ-0625) and hepatocellular carcinoma cell lines MHCC-97H (#MZ-2834), Huh7 (#MZ-2129), Hep3B (#MZ-2124), SNU-182 (#165735), and SNU-423 (#165738) were provided by MINZHOUBIO (Ningbo, China). Cells were maintained in Dulbecco's modified Eagle medium with 10% fetal bovine serum (Gibco, USA) and 1% penicillin/streptomycin and cultured in a humidified incubator containing 5% CO_2_ at 37°C.

### 2.3. Cell Transfection

The shRNAs against HLA-DQB1-AS1 and ZRANB2 and the corresponding negative control (sh-NC) were designed and synthesized by Ribobio Technology (Guangzhou, China). The cDNA of ZRANB2 was subcloned into the pIRES-hrGFP-1a vector (ABLIFe, Wuhan, China) for constructing the ZRANB2-overexpressing vector. The empty vector was used as the negative control. The transfection of shRNAs and ZRANB2-overexpressing vector and the corresponding negative controls was performed according to manufacturer's instructions using Lipofectamine RNA iMAX Reagent (Invitrogen, USA). After transfection for 24 h, cells were harvested.

### 2.4. Reverse Transcription Quantitative Polymerase Chain Reaction (RT-qPCR)

Total RNA was isolated from cells using TRIzol reagent (Tiangen Biotech, Beijing, China). A Hifair® II 1st Strand cDNA Synthesis SuperMix for qPCR (gDNA digester plus) (Yeasen, Shanghai) was used for the synthesis of cDNA. qPCR was performed with a Hieff UNICON® qPCR TaqMan Probe Master Mix (Yeasen) to analyze the gene expression of HLA-DQB1-AS1, ZRANB2, and GAPDH (the loading control) on a QuantStudio 7 Flex Real-Time PCR System (Applied Biosystems, USA). Relative gene expression of HLA-DQB1-AS1 and ZRANB2 was calculated with the 2^-*ΔΔ*Ct^ method and normalized by GAPDH. The qPCR primers (5′→3′) used in this study are listed as follows: HLA-DQB1-AS1, F: CTTGGGACCTGAGTAGACG and R: AATACACCCTGGACTCTGC; ZRANB2, F: GTTCTTCCAGTTCGCAGTC and R: GAGCTGGATCTTGATTTCGAC; and GAPDH, F: TCATTTCCTGGTATGACAACGA and R: GTCTTACTCCTTGGAGGCC.

### 2.5. Western Blotting

Proteins were separated on a sodium dodecyl sulfate-polyacrylamide gel and transferred onto polyvinylidene fluoride membranes (Bio-Rad, USA). After blocking with 5% bovine serum albumin for 50 min at room temperature, the membranes were incubated with primary antibodies against ZRANB2 (1 : 2000, ab82132, Abcam, Shanghai, China) and the loading control GAPDH (1 : 500, ab8245, Abcam) at 4°C overnight. The membranes were incubated with the horseradish peroxidase-labeled secondary antibodies for 1 h at room temperature in dark. Signal was detected using an enhanced chemiluminescence western blotting substrate (Bio-Rad, USA). The band density was quantified using the ImageJ software.

### 2.6. Proliferation Assessment

Colony formation assay was performed to assess cell proliferation. Transfected hepatocellular carcinoma cells were seeded into 6-well plates at a density of 1 × 10^3^ cells/well with the medium refreshed every 3 days. After 12 days, the medium was removed, and the cells were fixed with 4% paraformaldehyde for 30 min and stained with crystal violet for 30 min. After washing with PBS, cells were photographed, and the number of colonies was manually counted.

### 2.7. Apoptosis Assessment

Cell apoptosis was assessed with the TdT-mediated dUTP nick end labeling (TUNEL) method using a commercial kit (#40306ES20, Yeasen) according to the manufacturer's instructions. The hepatocellular carcinoma cells were grown in a 24-well plate at the density of 3 × 10^5^ cells per well and incubated for 24 h. Then, the 24-well plate was removed, and the medium was discarded. The cells were washed once with PBS and then fixed in 4% paraformaldehyde for 30 min. Then, cells were permeabilized in 0.1% Triton X-100 solution for 2 min on ice. After staining the cells with a TUNEL staining kit at 37°C for 1 h and 4,6-diamidino-2-phenylindole (DAPI; Solarbio, Beijing, China) for 10 min in the dark, cells were observed and photographed using a fluorescence microscope (Olympus, Japan). The number of apoptotic cells was manually counted as the average value in five randomly selected fields of view.

### 2.8. RNA Immunoprecipitation (RIP)

A Magna RIP RNA-Binding Protein Immunoprecipitation Kit (Millipore, USA) was used to assess the binding of HLA-DQB1-AS1 and ZRANB2 protein according to the manufacturer's recommendations. Cells were lysed with lysis buffer, and cell lysates were immunoprecipitated with anti-ZRANB2 (#A301-029A, Bethyl Laboratories, USA) at the concentration of 2 *μ*g/mg lysate and anti-immunoglobulin (IgG) antibodies (#ab6715, Abcam, Shanghai) at the concentration of 4 *μ*g/mg lysate. Immunoprecipitated RNA was extracted, purified, and reverse transcribed to cDNA. Relative enrichment of HLA-DQB1-AS1 was evaluated by RT-qPCR with the 2^−*ΔΔ*Ct^ methods as abovementioned. Anti-IgG was used as the negative control.

### 2.9. Statistical Methods

Difference comparison was performed by one-way analysis of variance using GraphPad Prism 9 (GraphPad, USA). All experimental data were derived from three independent assays and were expressed as the mean ± standard error of mean. *P* values less than 0.05 (two-tailed) were considered statistically significant.

## 3. Results

### 3.1. HLA-DQB1-AS1 Is Upregulated in Hepatocellular Carcinoma

**Figure 1:**
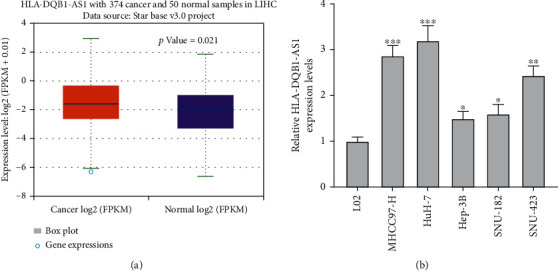
HLA-DQB1-AS1 is upregulated in hepatocellular carcinoma. (a) HLA-DQB1-AS1 expression, log2 (FPKM + 0.01) transformed, in 374 liver hepatocellular carcinoma (LIHC) tissues and 50 normal samples on EOCORI database. (b) RT-qPCR analysis of HLA-DQB1-AS1 expression in hepatocellular carcinoma cell lines and normal liver cell line LO2. ^∗^*P* < 0.05, ^∗∗^*P* < 0.01, and ^∗∗∗^*P* < 0.001.

According to ENCORI database [18], HLA-DQB1-AS1 exhibited increased expression by 1.68-fold change in 374 hepatocellular carcinoma tissues compared with 50 normal samples (*P* = 0.021, Figure 1(a)). HLA-DQB1-AS1 also showed upregulation in hepatocellular carcinoma cells than control normal liver cell line LO2 (Figure 1(b)). We explored the role of HLA-DQB1-AS1 using MHCC97-H and Huh-7 cells in subsequent assays since they showed the relatively high expression of HLA-DQB1-AS1.

### 3.2. HLA-DQB1-AS1 Deficiency Exerts Antiproliferative and Proapoptotic Effects on Hepatocellular Carcinoma Cells

**Figure 2:**
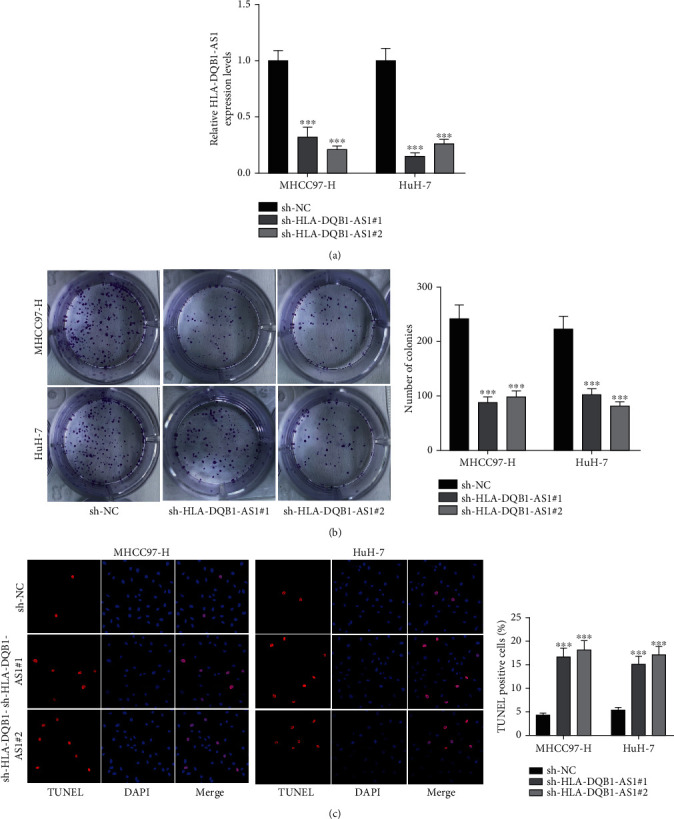
HLA-DQB1-AS1 deficiency exerts antiproliferative and proapoptotic effects on hepatocellular carcinoma cells. (a) RT-qPCR was performed to confirm the knockdown efficiency of HLA-DQB1-AS1. (b, c) Colony formation and TUNEL assays were performed to assess the proliferation and apoptosis of MHCC97-H and Huh-7 cells after sh-HLA-DQB1-AS1#1/2 transfection. ^∗∗∗^*P* < 0.001.

Transfection of sh-HLA-DQB1-AS1#1/2 significantly reduced HLA-DQB1-AS1 expression in MHCC97-H and Huh-7 cells (Figure 2(a)). sh-HLA-DQB1-AS1#1/2 causes a decrease in number of colonies and an increase in number of TUNEL-positive cells (Figures 2(b) and 2(c)), indicating that HLA-DQB1-AS1 knockdown promoted cell apoptosis and inhibited cell proliferation in hepatocellular carcinoma.

### 3.3. HLA-DQB1-AS1 Binds with ZRANB2 Protein

**Figure 3:**
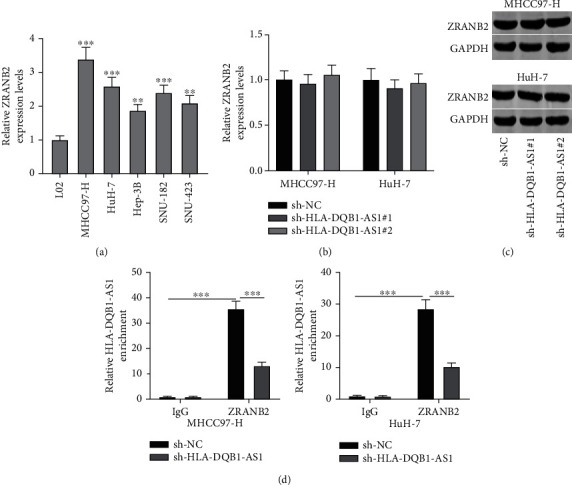
HLA-DQB1-AS1 binds with ZRANB2 protein. (a) RT-qPCR analysis of ZRANB2 expression in hepatocellular carcinoma cell lines and normal liver cell line LO2. (b, c) Effects of sh-HLA-DQB1-AS1#1/2 on ZRANB2 mRNA and protein expression, as assessed by RT-qPCR and western blotting. (d) RIP assay was performed to explore the interaction between HLA-DQB1-AS1 and ZRANB2 protein in MHCC97-H and Huh-7 cells after sh-HLA-DQB1-AS1 transfection. ^∗∗^*P* < 0.01; ^∗∗∗^*P* < 0.001.

Based on RBPDB database [19], HLA-DQB1-AS1 is predicted to bind with ZRANB2 protein. We detected ZRANB2 mRNA expression and demonstrated its upregulation in hepatocellular carcinoma cells (Figure 3(a)). Neither ZRANB2 mRNA nor ZRANB2 protein expression was affected by sh-HLA-DQB1-AS1 (Figures 3(b) and 3(c)). RIP assay was conducted, and its results confirmed the binding of HLA-DQB1-AS1 and ZRANB2 protein, which showed that HLA-DQB1-AS1 was enriched in the precipitates of anti-ZRANB2 compared with anti-IgG. However, enrichment of HLA-DQB1-AS1 in the anti-ZRANB2 was reduced in MHCC97-H and Huh-7 cells after transfection of sh-HLA-DQB1-AS1#1/2 (Figure 3(d)).

### 3.4. Bioinformatic Information of ZRANB2

**Figure 4:**
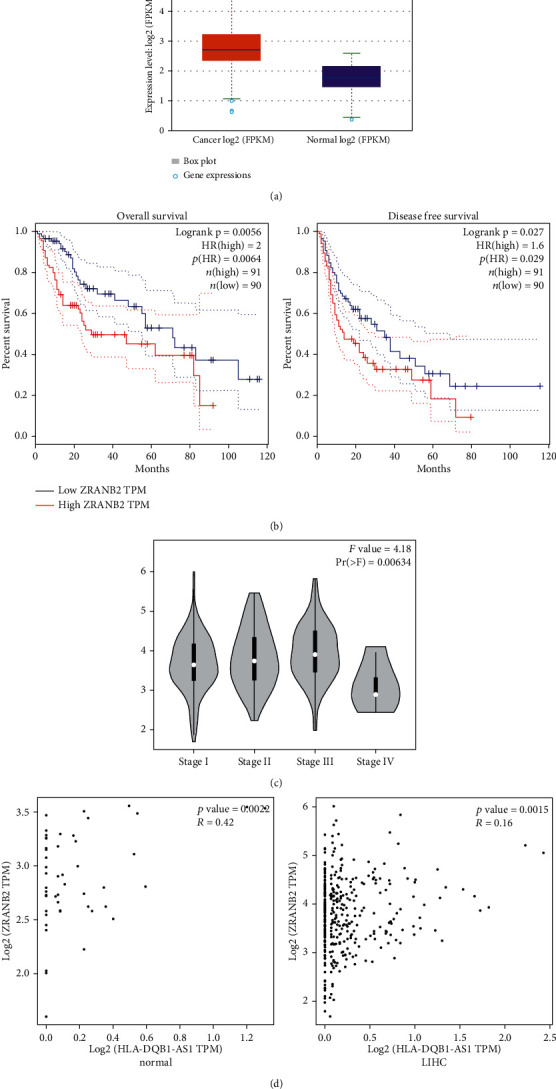
Bioinformatic information of ZRANB2. (a) ZRANB2 expression, log2 (FPKM + 0.01) transformed, in 374 LIHC tissues and 50 normal samples on EOCORI database. (b) Overall survival and disease-free survival curves of liver hepatocellular carcinoma patients with high or low expression of ZRANB2 were obtained from GEPIA database. (c) ZRANB2 expression in different stages of liver hepatocellular carcinoma was obtained from GEPIA database. (d) Expression correlation of HLA-DQB1-AS1 and ZRANB2 in liver hepatocellular carcinoma tissues and adjacent normal tissues was obtained from GEPIA database.

ZRANB2 was upregulated by 2.11-fold changes in 374 hepatocellular carcinoma tissues compared with 50 normal samples, as revealed by ENCORI database (*P* = 9.9*e* − 21, Figure 4(a)). Patients with higher ZRANB2 expression had a shorter overall survival and disease-free survival, which were derived from GEPIA database [20] (Figure 4(b)). Based on this database, ZRANB2 expression was also associated with stage of hepatocellular carcinoma. Its expression was higher in stage III and lower in stage IV than stage I (Figure 4(c)). The positive expression correlation between HLA-DQB1-AS1 and ZRANB2 was found significant in hepatocellular carcinoma adjacent normal tissues, while in hepatocellular carcinoma tissues, the expression correlation is slight (Figure 4(d)).

### 3.5. Proliferation of Hepatocellular Carcinoma Cells Was Suppressed by ZRANB2 Knockdown while Cell Apoptosis Was Enhanced

**Figure 5:**
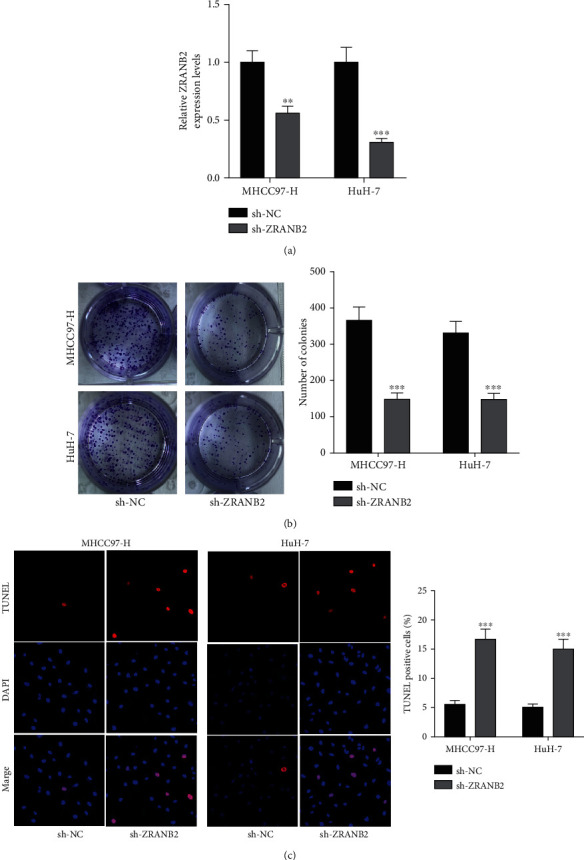
ZRANB2 knockdown suppressed proliferation and enhanced apoptosis of hepatocellular carcinoma. (a) RT-qPCR was performed to confirm the knockdown efficiency of ZRANB2. (b, c) Colony formation and TUNEL assays were performed to detect the proliferation and apoptosis in MHCC97-H and Huh-7 cells after sh-ZRANB2#1/2 transfection. ^∗∗^*P* < 0.01; ^∗∗∗^*P* < 0.001.

Transfection of sh-ZRANB2#1/2 caused a reduction in ZRANB2 expression in MHCC97-H and Huh-7 cells (Figure 5(a)). ZRANB2 knockdown reduced number of colonies and increased number of TUNEL-positive cells (Figures 5(b) and 5(c)), which suggested that ZRANB2 acted as an oncogene in hepatocellular carcinoma.

### 3.6. ZRANB2 Overexpression Rescued the Anticancer Effect of Silenced HLA-DQB1-AS1 on Hepatocellular Carcinoma Cells

**Figure 6:**
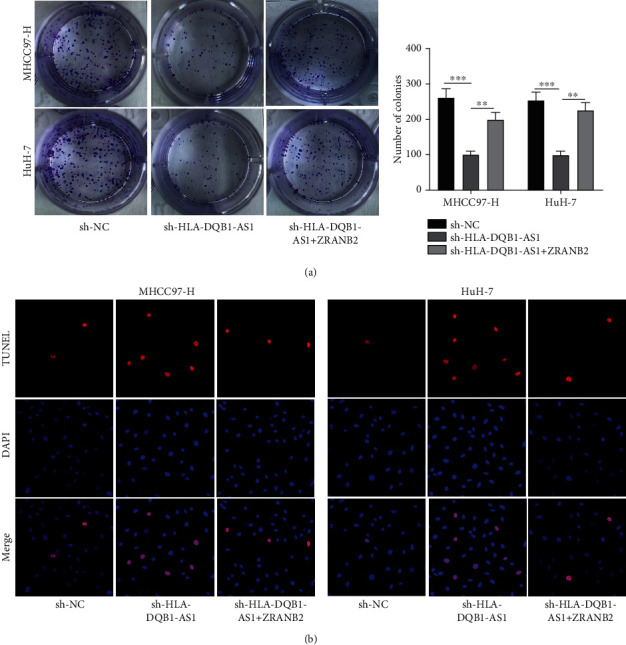
ZRANB2 overexpression rescued the anticancer effect of silenced HLA-DQB1-AS1. (b, c) Colony formation and TUNEL assays were performed in MHCC97-H and Huh-7 cells after transfection of sh-NC and sh-HLA-DQB1-AS1 and cotransfection of sh-HLA-DQB1-AS1+pIRES-hrGFP-1a-ZRANB2. ^∗∗^*P* < 0.01; ^∗∗∗^*P* < 0.001.

Results of colony formation and TUNEL assays indicated that ZRANB2 overexpression rescued the anticancer influences of HLA-DQB1-AS1 knockdown on the proliferation and apoptosis in MHCC97-H and Huh-7 cells (Figures 6(a) and 6(b)), suggesting that HLA-DQB1-AS1 exerted the tumor promoter role in hepatocellular carcinoma by the interaction with ZRANB2.

## 4. Discussion

Bioinformatic analysis revealed the upregulation of HLA-DQB1-AS1 in human hepatocellular carcinoma specimens. In our study, the upregulated expression of HLA-DQB1-AS1 in hepatocellular carcinoma cells compared with normal liver cell line was confirmed. Next, we detected the effects of HLA-DQB1-AS1 on the proliferation and apoptosis *in vitro* and identified the proproliferative and antiapoptotic effects of HLA-DQB1-AS1 on hepatocellular carcinoma cells, demonstrating the tumor promoter role of HLA-DQB1-AS1. HLA-DQB1 is the nearby gene of HLA-DQB1-AS1, and HLA-DQB1 alleles were associated with risk for hepatocellular carcinoma progression [21]. HLA-DQB1 Block2 CCCCC haplotype confers beneficial effects in hepatitis B virus-related hepatocellular carcinoma patients after hepatic resection [22]. lncRNAs can interact with RNA binding proteins to regulate hepatocellular carcinoma [23]. A representative example is that uroplakin 1A antisense RNA 1 promotes cell proliferation in hepatocellular carcinoma through interaction with enhancer of zeste 2 polycomb repressive complex 2 subunit [24]. Therefore, proteins that potentially bind with HLA-DQB1-AS1 were searched, and zinc finger RANBP2-type containing 2 (ZRANB2) was found by bioinformatic analysis. Our experimental results further confirmed the binding of HLA-DQB1-AS1 and ZRANB2 protein.

ZRANB2 is initially found in renal juxtaglomerular cells [25] and exists in the nucleus of human cells [26]. ZRANB2 has homologues among many species with the conservation in N-terminal end. It exhibits high expression in grade III ovarian serous papillary carcinoma [27]. The doxorubicin-induced accumulation of resistant breast cancer cells in the S phase is dependent on ZRANB2 isoform [28]. ZRANB2 shows upregulated expression in glioma in a clinical and cellular level. Its knockdown suppresses the proliferation and vasculogenic mimicry formation of glioma cells [29]. In our study, ZRANB2 was upregulated in hepatocellular carcinoma and its high expression predicts a poor prognosis of patients with this disease. The bioinformatic analysis revealed the close association of ZRANB2 with cancer stage of hepatocellular carcinoma. Its expression is higher in stage III and lower in stage IV than stage I. We revealed the upregulation of ZRANB2 in hepatocellular carcinoma cells and confirmed that ZRANB2 knockdown suppressed cell proliferation and enhanced cell apoptosis of hepatocellular carcinoma. Moreover, ZRANB2 overexpression rescued the anticancer effects of silenced HLA-DQB1-AS1 on proliferation and apoptosis of hepatocellular carcinoma cells, indicating that HLA-DQB1-AS1 exerts its oncogenic role in hepatocellular carcinoma cells by interaction with ZRANB2.

## 5. Conclusion

HLA-DQB1-AS1 promotes cell proliferation and inhibits apoptosis in hepatocellular carcinoma by the interaction with ZRANB2 protein. Our study not only innovatively demonstrates the oncogenic roles of HLA-DQB1-AS1 and ZRANB2 in hepatocellular carcinoma but also innovatively identifies the interaction between HLA-DQB1-AS1 and ZRANB2 protein, which enriches the molecular mechanism of the pathogenesis of hepatocellular carcinoma. Such conclusion needs to be confirmed in animal studies. In addition, more experiments are needed to explore the downstream mRNAs and/or signaling pathways of ZRANB2 protein in hepatocellular carcinoma.

## Data Availability

The data that support the findings of this study are available from the corresponding author upon reasonable request.
